# Significance of the entire C-terminus in biological activities mediated by the RON receptor tyrosine kinase and its oncogenic variant RON160

**DOI:** 10.1186/1756-9966-27-55

**Published:** 2008-10-25

**Authors:** Yi Lu, Hang-Ping Yao, Ming-Hai Wang

**Affiliations:** 1 Laboratory of Cancer Biology and Therapeutics, First Affiliated Hospital, Zhejiang University School of Medicine, Hangzhou, Zhejiang 310003, PR China; 2 Cancer Biology Center and Department of Pharmaceutical Sciences, School of Pharmacy, Texas Tech University Health Sciences Center, Amarillo, TX 79106, USA; 3 Institute of Infectious Diseases, First Affiliated Hospital, Zhejiang University School of Medicine, Hangzhou, Zhejiang 310003, PR China; 4 Department of Pharmaceutical Sciences, School of Pharmacy, Texas Tech University Health Sciences, Amarillo, TX 79106, USA

## Abstract

The RON receptor tyrosine kinase regulates epithelial cell homeostasis and tumorigenesis by transducing multiple signals through its functional domains. The present study was to determine the significance of the entire C-terminus in RON or its variant RON160-mediated activities related to cell motility and tumorigenesis. Analysis of protein phosphorylation revealed that elimination of the entire C-terminus significantly impairs the ligand-dependent or independent RON or RON160 phosphorylation and dimerization. Phosphorylation of downstream signaling proteins such as Erk1/2, AKT, and p38 MAP kinase was also diminished in cells expressing the C-terminus-free RON or RON160. These dysfunctional activities were accompanied with the inability of truncated RON or RON160 to mediate cytoplasmic β-catenin accumulation. Functional analysis further demonstrated that truncation of the C-terminus significantly impairs RON or RON160-mediated cell proliferation, morphological changes, and cellular migration. Significantly, oncogenic RON160-mediated tumor growth in athymic nude mice was lost after the deletion of the C-terminus. Thus, the C-terminus is a critical component of the RON receptor. The entire C-terminus is required for RON or RON160-mediated intracellular signaling events leading to various cellular activities.

## Introduction

The RON (Recepteur d'Origine Nantaise) receptor tyrosine kinase is the product of the c-RON proto-oncogene [1] and the high affinity receptor for macrophage-stimulating protein (MSP) [2,3], also known as hepatocyte growth factor like protein [4]. RON is mainly expressed in cells of epithelial origin and required for embryonic development [5,6]. Biochemically, RON is first synthesized as a single-chain precursor with 1400 amino acids [1-3]. Proteolytic conversion of pro-RON into a mature RON is a step necessary for MSP binding and biological activities [1-3]. This process is mediated by proteases at the cleavage site of Pro^304^-Lys^305^, resulting in a 180 kDa heterodimeric protein with a 40 kDa extracellular α-chain and a 145 kDa transmembrane β-chain [1-3], both chains are linked by a disulphide bound [1-3]. Several functional domains have been identified in the RON protein. In the RON β-chain, the extracellular sequences contain a N-terminal semaphoring (Sema) domain followed by a plexin-semaphorin-integrin (PSI) motif and four IPT (Immunoglobulin, Plexin, and Transcription factor) domains [1,7]. The sema domain is responsible for MSP binding and receptor dimerization [8]. The IPT domains are essential in regulating RON maturation and kinase activities [9,10]. Deletion of the first or fourth IPT domain through the mRNA splicing processes has been linked to constitutive RON phosphorylation and impaired receptor maturation [9,11]. The juxtamembrane domain, tyrosine kinase domain and C-terminus are three functional structures in the intracellular sequences of the RON β-chain [1,7]. Deletion of the juxtamembrane domain has been shown to enhance RON phosphorylation [12]. In contrast, deletion of last 46 amino acids in the kinase domain severely impairs MSP-induced RON phosphorylation and tyrosine kinase activities [13]. The C-terminus contains a bidentate motif (-Y^1353^VQLPATY^1360^MNL-) and additional determinants [14,15]. Both positive and negative effects of the C-terminus have been demonstrated by *in vitro *and *in vivo *studies [14-16]. Thus, the functional domains are critically important in regulating RON-mediated activities.

Studies of RON in epithelial carcinogenesis have recently demonstrated that RON is highly expressed in various primary tumor samples including breast, colon, and pancreatic cancers [17-19]. Overexpression has also been shown to be associated with advanced clinical stages and poor clinical outcomes [20-22]. One pathogenic feature associated with RON overexpression is the production of biologically active RON variants [23]. These variants are produced either by alternative mRNA splicing or by alternative mRNA initiation [24]. A typical example is RON160 identified in primary colon cancer samples and in established cell lines [11,23]. RON160 is derived from an mRNA splicing transcript that has an in-frame deletion of 109 amino acids coded by exons 5 and 6. These 109 amino acids constitute the first IPT domain in the RON β-chain extracellular sequences [7,11]. RON160 has a molecular mass of 160 kDa and is constitutively phosphorylated with increased kinase activities [11]. High levels of Erk1/2, PI-3 kinase, and AKT activities are often observed in RON160 expressing cells [11,23]. Expression of RON160 results in transforming phenotypes in rodent fibroblast and human colonic epithelial cells and causes tumor growth in athymic nude mice [11,23-25]. Moreover, RON160 stimulates colonic cell morphological changes and motilities characterized by epithelial to mesenchymal transition (EMT) [25-27]. Although the mechanisms underlying RON160 mediated tumorigenesis are currently unknown, it is believed that the tumorigenic potential is attributed to its increased kinase activities leading to enhanced intracellular signaling cascades. Thus, RON overexpression, accompanied with generation of tumorigenic RON variants, is a pathogenic factor contributing to pathogenesis of various epithelial cells.

The present study was to determine the significance of the entire C-terminus in regulating RON or RON160-mediated biological activities. Previous studies have showed that the C-terminal bidentate motif is essential in regulating RON kinase activities leading to increased cellular functions [14,16]. However, biochemical analysis suggests that the C-terminus plays a negative role in regulating RON kinase activities [15]. This is evident in experiments showing that the C-terminus and the peptide containing Y^1353^/Y^1630 ^or substituted F^1353^/F^1360 ^motif strongly inhibit RON kinase activities [15]. In contrast, experiments of tumorigenesis mediated by certain RON mutants suggest that the C-terminal Y^1353^/Y^1360 ^motif is not required for tumorigenesis [28]. The RON mutant (M^1254^T) with F^1353^/F^1360 ^substitutions is capable of mediating tumor growth in nude mice, which is comparable to the control RON mutant without Y^1353^/Y^1360 ^substitution [28]. Considering the pathogenic significance of RON160 in colonic epithelial cells, we wanted to determine the importance of the entire C-terminus in RON or RON160-mediated activities. By generating cDNA encoding human RON or RON160 free of the C-terminus, we demonstrated that the entire C-terminus is critically important in regulating RON/RON160 kinase activities and essential for RON or RON160-mediated biological activities.

## Materials and methods

### Cells and reagents

NIH3T3 cells expressing RON or RONΔ160 were used as previously described [23]. Mouse monoclonal antibodies (mAb) Zt/g4 and Zt/c1 specific to RON extracellular sequences) and rabbit polyclonal IgG antibodies (R*5029, specific to the RON C-terminal peptide) were used as previously described [23,29]. Mouse anti-phosphotyrosine (PY-100), goat or rabbit IgG antibodies specific to regular or phosphor- Erk1/2 (p44/42), p38 MAP kinase, AKT, GSK-3β, and β-catenin were from Cell Signaling Inc (Beverly, MA). Normal mouse IgG and goat anti-mouse IgG conjugated with FITC were from Jackson Laboratories (Maine).

### Generation and expression of RON or RON160 variant free of the C-terminal tail

The RON C-terminus contains 55 amino acids starting from Ser^1346^, the first amino acids after the tyrosine kinase domain, and ending at Thr^1400^, the last amino acid of RON [1,7]. Using RON or RON160 cDNA as the template, the RON C-terminus-free (cf) and RON160-cf cDNA free of the entire C-terminal sequences were generated by PCR techniques and confirmed by DNA sequence analysis. The cDNA was inserted into the expression vector pcDNA3.1 (Invitrogen) and stable NIH3T3 cells expressing RON-cf or RON160-cf were established by DNA transfection techniques [23]. Positive cells were isolated by incubation with anti-RON mAb Zt/c1 followed by magnetic beads conjugated with goat-anti-mouse IgG as previously described [29].

### Immunoprecipitation and Western blot analysis

Immunoprecipitation of RON or other proteins from cellular lysates was performed using Zt/c1 or other mAb followed by Western blot analysis as previously described [23]. Briefly, cells (3 × 10^6 ^cells/sample) were lysed in the lysis buffer as previously described [23]. Cellular proteins were immunoprecipitated overnight with Zt/g4 or other antibodies (2 μg mAb per sample) coupled with protein G Sepharose beads. After washing, samples were boiled at 100°C for 4 min and then separated in 7% SDS-PAGE under reduced condition. The proteins were transferred into the membrane and blocked with 1% BSA in TBS-T buffer. Western blot analysis was carried out using rabbit IgG anti-RON or other specific antibodies followed by HRP-conjugated second antibodies. The reaction was developed with enhanced ECL reagents and analyzed by the VersaDoc imagine system (Bio-Rad).

### Biotinylation of cell surface protein

Due to lack of antibodies to detect RON-cf and RON160-cf in Western blotting, the method of protein biotinylation was used to label RON-cf or RON160-cf on cell surface (CalBiochem). Biotin-labeled proteins were immunoprecipitated with mAb Zt/c1 and detected by HPR-conjugated avidin in Western blot analysis.

### Cell proliferation assays

T3-RON, -RON-cf, -RON160, or -RON160-cf cells (0.8 × 10^4 ^cells/well) were seeded in a 96-well plate in triplicate in DMEM with 5% FBS. NIH3T3 cells were used as the control. RON agonistic mAb Zt/g4 (2 nM) was added simultaneously after initiation of cell culture [29]. After incubation for 5 days, the number of cells was counted as previously described [23].

### Cell surface immunofluorescent analysis

3T3-RON, -RON-cf, -RON160 and -RON160-cf cells were incubated with mAb Zt/c1 (2 μg mAb per sample) followed by FITC-coupled goat anti-mouse IgG. 3T3-RON cells with normal mouse IgG served as the negative control. Labeled samples were analyzed for fluorescent intensities by FACScan as previously described [29].

### Assays for cell morphological changes and migration

3T3-RON, -RON-cf, -RON160 or -RON160-cf cells (1 × 10^5 ^cells/well) were incubated in a 24-well plate, stimulated with or without mAb Zt/g4 (2 nM) for 5 days, and then photographed (magnification × 200). Parental NIH3T3 cells were used as the control. The migration assay was carried out as detailed previously [23]. Briefly, cell monolayers were wounded by a plastic tip and then treated with Zt/g4 (2 nM). After incubation for 48 h, migrated cells in the wounded area were photographed and measured.

### *In vivo *tumorigenic assays

Experiments were performed as previously described [23]. The use of animals was apprived by the institutional animal usage committee of the Texas Tech University Health Sciences Center (Approval number 0421). Briefly, 3T3-RON160 or -RON160-cf cells were inoculated subcutaneously into the posterior flank of athymic nude mice (1 × 10^6 ^cells per mouse in 0.2 ml PBS, three mice per group). The tumor growth was monitored daily for 30 days. Parental NIH3T3 and 3T3-RON cells were used as the control. The latency was determined as the period of time required by tumors to reach a diameter of 0.5 cm [23].

### Statistical analysis

Differences between control and experimental groups were determined by student *t *test. The statistical differences at P < 0.05 were considered significant.

## Results

### Generation and expression of RON-cf and RON160-cf in NIH-3T3 cells

**Figure 1:**
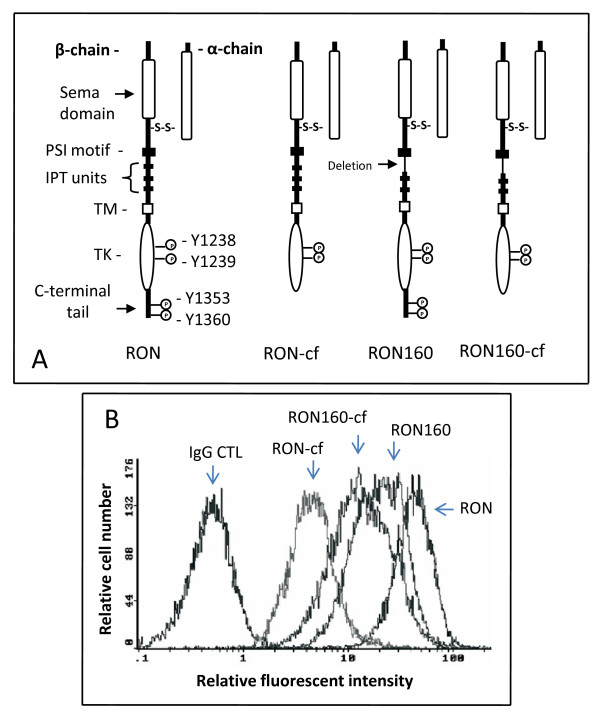
**Generation of C-terminus-free RON and RON160 and their expression in NIH3T3 cells**: (A) Schematic representation of the RON, RON160 and their C-terminus-free variants. RON is composed of a 40 kDa α-chain and a 150 kDa β-chain. The β-chain has several domains including a sema domain, a PSI motif, four IPT units, followed by a transmembrane (TM) domain, a juxtamembrane domain, a tyrosine kinase (TK) domain, and a short C-terminal tail. The generation of C-terminus-free RON or RON160 variants was carried out as detailed in Materials and Methods. (B) Cells (1 × 10^5 ^cells/sample) were incubated for 45 min at 4°C with mAb Zt/g4 (1 μg/sample) or normal mouse IgG (1 μg/sample) followed by goat anti-mouse IgG coupled with FITC. Immunofluorescent intensities were measured by FACSan. One of two experiments with similar results.

Previous studies have shown that F^1353^/F^1360 ^substitutions in the C-terminal bidentate motif do not affect MSP-induced RON phosphorylation but impair cell migration [16]. Other studies have found that the C-terminal tail negatively regulates RON kinase activities [15]. To address the importance of the entire C-terminus in RON or RON160-mediated biological activities, the cDNA encoding RON or RON160 free of the C-terminus was generated by PCR techniques that eliminate the last 55 amino acids (from Ser^1346 ^to Thr^1400^) in the RON protein. The schematic representation of RON, RON160, RON-cf, and RON160-cf was presented in Fig. 1A. The DNA sequence analysis confirmed that the truncated cDNA were corrected generated as designed (data not shown). Upon selection of stably transfected 3T3 cell lines, the expression of RON-cf or RON160-cf, along with RON or RON160, was analyzed by the immunofluorescent cell surface analysis. Results in Fig. 1B showed expression of RON-cf and RON160-cf on the cell surface as evident by Zt/g4 immunofluorescent detection. The levels of RON-cf or RON160-cf were relatively lower than those of RON or RON160 in expressed cells. These results suggest that elimination of the c-terminus has no effect in the process of RON or RON160 for the cell surface localization.

### The C-terminal tail is required for spontaneous or induced phosphorylation of RON or RON160

**Figure 2:**
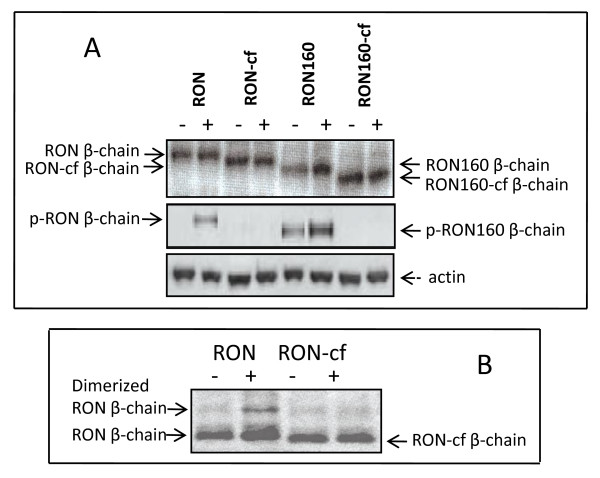
**Effect of the C-terminal truncation on agonistic mAb Zt/g4-induced RON or RON160 phosphorylation and dimerization**. (A) 3T3-RON or other cells (4 × 10^6 ^cells/sample) were stimulated with or without mAb Zt/g4 (2 nM) in serum-free conditions for 10 min. Half of the cells were biotinylated for cell surface proteins. Labeled RON, RON160, or their variants were immunoprecipitated with mAb Zt/c1 and detected in Western blotting using avidin-conjugated antibodies (top panel). The other half of the cells were lysed and immunoprecipitated by Zt/c1. Phosphorylated proteins were detected by mAb PY-100 (middle panel). To ensure equal amounts of samples used, cell lysates were directly analyzed for actin in Western blotting (bottom panel). (B) Cells were stimulated as above and then treated with a permeable cross-linker followed by cell surface biotinylation [13]. The proteins were immunoprecipitated and detected in Western blot analysis as described in (A). Data shown here are from one of three experiments with similar results.

To determine if the truncation of the C-terminus affects RON or RON160 expression and phosphorylation, Western blotting was first performed to determine the sizes of RON-cf or RON160-cf. Results in Fig. 2A (top panel) showed the correct sizes of RON-cf and RON160-cf. Using these cell lines, the effect of the C-terminal truncation on RON or RON160 phosphorylation was determined using the RON agonistic mAb Zt/g4 as the stimulant. Zt/g4 has agonistic activities and is more potent than MSP [29]. As shown Fig. 2A (middle panel), Zt/g4 induced RON phosphorylation in 3T3-RON cells but had no effect on 3T3-RON-cf cells. In 3T3-RON160 cells, spontaneous phosphorylation was seen and further enhanced by Zt/g4 stimulation. However, these effects were not observed in RON160-cf cells. Similar results were also seen when cells were stimulated with MSP (data not shown).

To address if the C-terminus was involved in RON dimerization, a step required for phosphorylation, cells were stimulated with Zt/g4 followed by cross-linking and Western blot analysis. RON dimerization was seen in Zt/g4 stimulated cells evident by the appearance of high molecular bands, but not in quiescent cells. Stimulation of RON-cf did not cause any visible dimerization (Fig. 2B). Similar results were also seen in RON160-cf cells (data not shown). These results, together with those in Fig. 2A, suggest that the C-terminus was required for ligand-dependent or independent phosphorylation of RON or RON160.

### Effect of C-terminal truncation on RON/RON160-mediated activation of three signaling pathways

**Figure 3:**
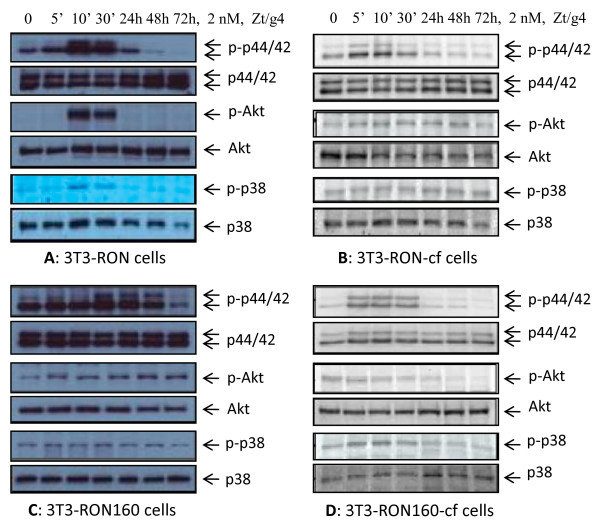
**Effect of the C-terminal truncation on RON or RON160-mediated activation of downstream signaling proteins**: Cells (2 × 10^6 ^cells/sample) were stimulated with RON agonistic mAb Zt/g4 (2 nM) in serum-free conditions for various times as indicated. Proteins (50 μg per lane) from cell lysates were subjected to Western blot analysis using antibodies specific to regular or phospho-p44/42, Akt, and p38 MAP kinase, respectively. Data shown here are from one of three experiments with similar results.

To determine if the C-terminus was involved in RON or RON160-mediated downstream signaling events, phosphorylation of Erk1/2, Akt, and p38 MAP kinase was studied in 3T3-RON-cf and RON160-cf cells. As shown in Fig. 3A, Zt/g4 stimulation of 3T3-RON cells induced strong Erk1/2 phosphorylation in a time-dependent manner. High levels of AKT phosphorylation were also seen. The levels of p38 MAKP kinase phosphorylation were relatively low. In 3T3-RON160-cf cells, truncation of the C-terminus significantly reduces the Zt/g4-induced RON-mediated Erk1/2 phosphorylation although low levels of phosphorylation were still visible. Trance amounts of Akt phosphorylation were also seen in 3T3-RON-cf cells. Phosphorylation of p38 MAP kinase was at the minimal level. In the case of RON160, spontaneous phosphorylation of Erk1/2, Akt, and p38 MAP kinase was observed in 3T3-RON160 cells (Fig. 3C). Zt/g4 stimulation further enhanced the levels of phosphorylation. In contrast, elimination of the C-terminus significantly reduced the phosphorylation levels of Erk1/2, Akt, and p38 MAP kinase in response to Zt/g4 stimulation. As shown in Fig. 3D, the levels of Erk1/2 phosphorylation, although still visible, were dramatically reduced. Similarly, phosphorylation of Akt and p38 was at the minimal level. These results, together with those from Fig. 2, demonstrate that the C-terminus is not only important for induced RON phosphorylation and spontaneous RON160 auto-phosphorylation, but also essential for activation of downstream signaling pathways. Moreover, truncation of the c-terminus was unable to completely eliminate Erk1/2 phosphorylation.

### The C-terminal tail is critical in regulating RON or RON-160-mediated β-catenin expression

**Figure 4:**
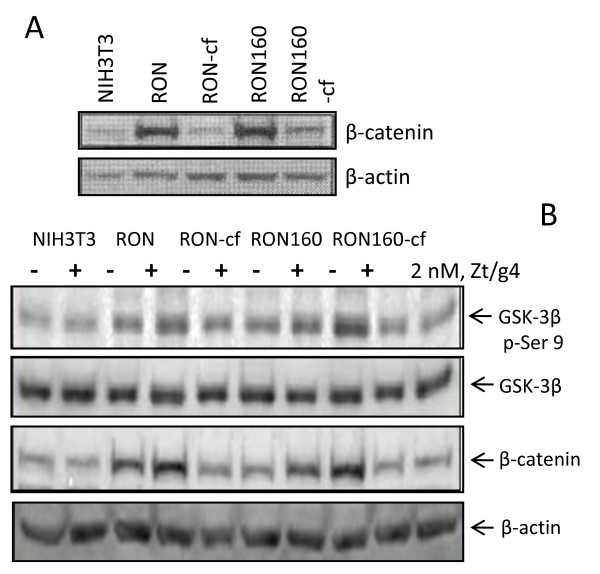
**Effect of the C-terminal truncation on RON or RON160-mediated cytoplasmic accumulation of β-catenin**: (A) Cellular proteins (50 μg/lane) from individual cell lines were subjected to Western blot analysis using rabbit IgG antibodies to β-catenin. (B) Cells were stimulated with Zt/g4 (2 nM) for 30 min. Cellular proteins were subjected to Western blot analysis using mouse IgG mAb against phosphor-Ser-9 of GSK-3β or regular GSK-3β. Expression of β-catenin was also determined. β-actin was probed as the loading controls. Data shown here are from one of three experiments with similar results.

Cytoplasmic β-catenin accumulation is regulated by GSK-3β (16). RON or RON160 inactivates GSK-3β by increasing its Ser-9 phosphorylation (17,18). We wanted to determine if elimination of the C-terminus affects RON or RON160-meidated β-catenin accumulation. As shown in Fig. 4A, increased β-catenin expression was observed in RON or RON160 cells in comparison with parental 3T3 cells. However, this effect was not seen in RON-cf and RON160-cf cells. A slight increase in β-catenin was found in 3T3-RON160-cf cells but hardly detected in RON-cf cells. In analyzing GSK-3β Ser-9 phosphorylation, we found that Zt/g4 increased GSK-3β Ser-9 phosphorylation in both 3T3-RON and RON160 cells. However, this effect was not seen in cells expressing RON-cf or RON160-cfs (Fig. 4B). The stimulating effects of Zt/g4 on increased expression of β-catenin in 3T3-RON or RON160 cells but not in 3T3-RON-cf or RON160-cf cells were also observed in Fig. 4B. Thus, the C-terminus is important in RON/RON160-mediated GSK-3β Ser-9 phosphorylation leading to increased stability of β-catenin in the cytoplasm.

### Deletion of the C-terminus is sufficient to abolish RON or RON160-mediated cell proliferation, morphological change, and migration

**Figure 5:**
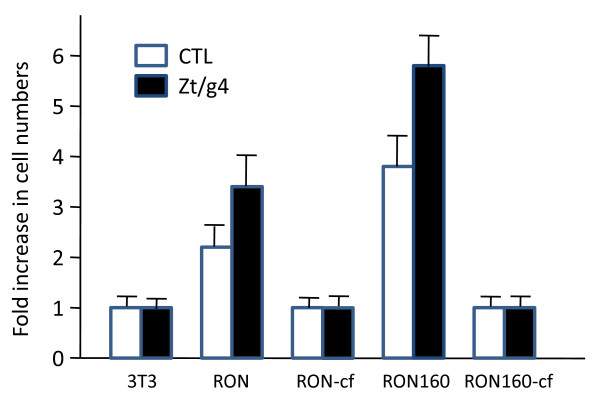
**Effect of C-terminal truncation on RON-RON160-mediated cell proliferation**: NIH3T3 cells expressing RON, RON160 or others (1 × 10^5^cells/well) were cultured in triplicate in a 96-well plate in DMEM with 5% FBS. Cells were stimulated with 2 nM of mAb Zt/g4 for 5 days. Cell numbers were determined as previously described [11]. Parental NIH3T3 cells were used as the control. Results shown here are from one of three experiments with similar results.

Overexpression of RON or RON160 in NIH3T3 cells resulted in increased cell proliferation, which was further enhanced after cells were stimulated with Zt/g4 (Fig 5). Similar results were also seen after MSP stimulation (data not shown). However, the spontaneous and Zt/g4-induced growth activities were completely lost in cells which expressed RON-cf or RON160-cf. In both cases, the numbers of cells were at the levels relatively comparable to those of control NIH-3T3 cells. It needs to point out that the low levels of Erk1/2 activation in RON-cf or RON160-cf cells as shown in Fig. 3B and 3D were not sufficient to cause proliferation of RON-cf and RON160-cf cells.

**Figure 6:**
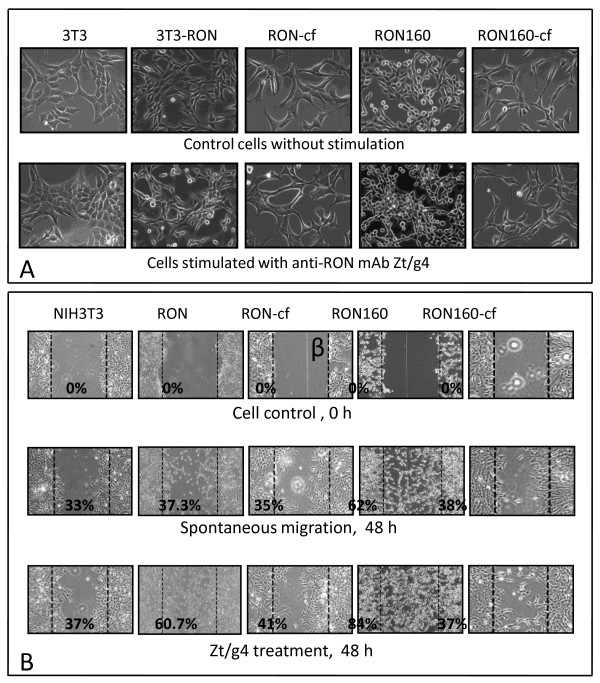
**Effect of C-terminal truncation on RON or RON160-mediated cell morphological change and migration**: (A) Cell incubation and Zt/g4 stimulation were carried out as detailed in Fig. 5. Three days after stimulation, cells were photographed for morphological changes as previously described [23]. (B) Cell migration was determined as detailed in Materials and Methods. Results shown here are from one of three experiments with similar results.

Expression of RON160 but not RON often resulted in NIH3T3 cell morphological changes such as the round up appearance. These changes were not seen in cells expressing RON160-cf (Fig. 6A, top panel). Upon stimulation with Zt/g4, cell shape changes were observed in 3T3-RON cells. However, this effect was not seen in 3T3-RON-cf cells (Fig. 6A, bottom panel). Stimulation of 3T3-RON160 cells with Zt/g4 further changed cell morphologies due to the formation of focus-like cell clusters. However, these activities were not present in 3T3-RON160-cf cells (Fig. 6A, bottom panel). In all cases, 3T3-RON-cf and 3T3-RON160-cf cells displayed typical fibroblast morphologies similar to those shown by parental NIH-3T3 cells.

Spontaneous cell migration was increased in cells expressing RON or RON160 (Fig. 6B). However, this effect was not observed in 3T3-RON-cf and 3T3-RON160-cf cells. Stimulation of 3T3-RON or -RON160 cells with Zt/g4 further enhanced cell migration. More than 70% of the open space was covered by migrated 3T3-RON or -RON160 cells. In contrast, the migration of 3T3-RON-cf or RON160-cf cells upon Zt/g4 treatment remained at the levels comparable to the control 3T3 cells. These results, together with those from Fig. 5 and 6A, demonstrate that elimination of the C-terminus significantly diminishes the ability of RON or RON160 to mediate cell proliferation, morphological changes, and migration.

### Inability of C-terminus-free RON160 to mediate tumor growth in Balb/c mice

RON160 is the oncogenic variant that initiates and promotes tumor growth when transfected cells were inoculated into mice (Table 1). NIH3T3 cells expressing RON do not cause tumor growth. To determine if elimination of the C-terminus affects the RON160-mediated tumor formation, 3T3-RON160-cf cells were inoculated into athymic nude mice and tumor growth was monitored. Results in Table 1 show that NIH3T3 cells did not form tumor in mice as expected. 3T3-RON160 cells formed tumors in all 3-injected mice with a latency of 9 days. The average sizes of tumors were 2.6 × 2.1 cm in diameter. No tumor formation was observed in mice inoculated with 3T3-RON160-cf, indicating that RON160-cf loses the ability to initiate tumor growth in mice.

**Table 1:** Effect of the C-terminal truncation on RON160-mediated tumor growth in athymic nude mice*

**Cells**	**Mice with tumor/total mice**	**Onset of tumor mass (days)**	**Size of tumor (L × W, cm)**	**Latency (days)**
NIH3T3	0/3	**-**	-	-

3T3-RON	0/3	**-**	**-**	**-**

3T3-RON-cf	0/3	**-**	**-**	**-**

3T3-RON160	3/3	19	2.6 × 2.1	9

3T3-RON160-cf	0/3	-	-	-

*Cells (1 × 10^6 ^cells in 0.1 ml PBS) were subcutaneously injected into the posterior flank of a female athymic nude mouse (3 mice per group). The mice were monitored daily for tumor growth at the site of inoculation. Animals in which tumor did not form were observed for additional 1 week. The latency was determined as the period of time required by tumors to reach a diameter of 0.5 cm. Results were from one of two experiments with similar outcomes.

## Discussion

The purpose of this study was to determine the significance of the entire C-terminus in RON or RON160-mediated biological activities. The C-terminus plays the vital role in regulating RON-mediated activities. Both positive and negative activities have been documented [14,15], suggesting that the functions of the C-terminus are complex. Studies from previous reports have mainly focused on identification and characterization of structural domains in the C-terminus [14,16,28]. The role of the entire C-terminus was often ignored or less emphasized. For example, the sequence of -Y^1353^-VQLPAT-Y^1360^MNL- designated as the bidentate domain in the C-terminus has been studied in details [14,16,28]. The domain is known as the docking site for anchoring intracellular proteins necessary for transduction of RON signals [14,16,28]. However, this domain has the inhibitory effect on RON kinase activities [16] and the inhibitory activity remains strong even Y^1353^/Y^1360 ^was substituted with other amino acids [16]. This suggests the existence of additional determinants in the C-terminus [16]. We took a function-based approach to study the roles of the entire C-terminus in RON or RON160-mediated biological activities. Our data demonstrated that the C-terminus is required for ligand-dependent RON phosphorylation, dimerization, activation of Erk1/2 and AKT, and cellular activities such as cell proliferation and migration. The C-terminus was also critical for ligand-independent RON160 phosphorylation and downstream signaling events. Significantly, elimination of the entire C-terminus completely blocked the RON160-mediated tumor growth in athymic nude mice.

The RON receptor contains several functional domains critical in ligand binding, protein maturation, and biological activities [1,7,24]. As shown in RON160, deletion of the first IPT domain in the extracellular sequence of the RON β-chain results in the gain of oncogenic functions [11,23,29]. In contrast, splicing out the last 46 amino acids coded by exon 19 in the catalytic kinase domain creates a kinase-dead variant RON170, which is unable to transduce signals [13]. Previous studies have defined two functions of the C-terminal tail. One is to act as a docking site for interaction with intracellular signaling proteins [14]. This is mainly manifested by the bidentate tyrosine motif [14]. Upon phosphorylation of Y^1353^/Y^1360^, the motif serves as an anchor to recruit downstream signaling molecules such as PI-3 kinase [16], Grb-2 [30], and others [31]. Substitutions of Y^1353^/Y^1360 ^with phenylalanine significantly impair RON-mediated cell migration and other activities but have no effect on MSP-induced RON phosphorylation at Y^1238^/Y^1239 ^in the kinase domain [16]. Another function of the C-terminus is the auto-inhibitory effect on the RON kinase activities [15]. This activity is presumably mediated by functional domains in the C-terminus that interact with the kinase catalytic domain [15]. There is evidence suggesting that the bidentate motif is involved in interaction with the RON kinase domain. However, substitutions of Y^1353^/Y^1360 ^cannot eliminate this effect, indicating that other determinants in the C-terminus are also involved in the inhibitory effect [15]. The results from our current studies showed that the entire C-terminus is essential in RON auto-phosphorylation and signal transduction. First, deletion of the C-terminus resulted in the inability of RON to undergo tyrosine phosphorylation upon ligand or agonistic mAb stimulation. Spontaneous and Zt/g4-induced phosphorylation of RON160 were also abolished. These data suggested that ligand-dependent or independent RON or RON160 phosphorylation occurred only in the presence of the C-terminus. These findings are different from previous studies showing that ligand induces RON phosphorylation in the presence of F^1353^/F^1360^. Second, activation of downstream signaling proteins such as Erk1/2 and ATK was diminished in cells stably expressing RON-cf. This effect occurred also in RON160-cf cells. As shown in Fig. 6C and 6D, high levels of spontaneous and Zt/g4-enhanced Erk1/2 or ATK phosphorylation were dramatically reduced in RON160-cf cells, confirming that the entire C-terminus is essential in activation of downstream signaling components. However, it needs to point out that truncation of the entire C-terminus cannot completely abolish MSP or Zt/g4-induced phosphorylation of Erk1/2 and other signaling proteins as shown in Fig. 6B and 6D. It is possible that activated RON kinase by itself is capable of activating downstream signaling events although the levels of the activation are relatively low. Finally, the increased stabilization by RON or RON160 of cytoplasmic β-catenin was significantly impaired due to the truncation of the C-terminus. The impairment was also accompanied by the inability of RON-cf or RON160 -cf in response to Zt/g4 induced GSK-3β Ser-9 phosphorylation. Cytoplasmic β-catenin accumulation plays a role in RON or its variant-mediated tumorigenic activities in colonic epithelial cells [26]. In conclusion, the entire C-terminus plays a pivotal role in controlling RON or RON160-mediated phosphorylation and signaling events.

The role of the C-terminus in RON or oncogenic RON variant-mediated tumorigenic activities is complex. Most of the studies focused on the bidentate tyrosine Y^1353^/Y^1360 ^[14,28]. Analysis of oncogenic mutant RON^D1232V ^has revealed that substitution of Y^1353^/Y^1360 ^completely abolishes *in vitro *cell-transforming activities and tumor growth in animal models [28,32]. However, this effect was not seen in cells expressing oncogenic mutant RON^M1254T ^[28,32]. RON^M1254T ^continues to cause cell transformation and tumor growth even the C-terminal Y^1353^/Y^1360 ^were substituted. Nevertheless, Y^1353^/Y^1360 ^substitutions have a negative impact on RON^M1254T^- mediated tumorigenic activities. As shown in the focus formation and tumor growth assays, the numbers of foci was reduced and the tumor latency was prolonged [28]. These results imply that the bidentate motif is involved in the oncogenic activity of RON^M1254T ^but is displaceable. Further studies demonstrated that the catalytic kinase activities play the vital role in these pathological events [28,32,33]. Considering the report showing that the synthetic peptide containing Y^1353^/Y^1360 ^or even F^1353^/F^1360 ^inhibits RON kinase activities both *in vitro *and *in vivo *[15], it is likely that the requirement of the bidentate motif in oncogenic RON mutant-mediated activities depends largely on the activation status of the kinase domain. We demonstrated that the entire C-terminus is required for RON-mediated cell growth, migration, and morphological changes. The C-terminus was also crucial for RON160-mediated cellular transformation and tumorigenic growth *in vivo*. The function of the entire C-terminus, in the case of RON and RON160, is not displaceable. As shown in results, increased cell proliferation, morphological change, and enhanced migration were diminished in cells expressing RON-cf and RON160-cf. These changes were directly associated with the inability of RON-cf or RON160-cf to undergo tyrosine phosphorylation and to activate the high levels of the downstream signaling events. Thus, the C-terminus is an intriguing component in the RON protein. In quiescent cells, it controls RON kinase activities through the use of the bidentate motif to interact with the catalytic domain [15]. Such interaction seems to be necessary in maintaining RON in the inactive mode. Upon ligand stimulation and subsequent phosphorylation at Y^1353^/Y^1360^, the C-terminal tail is released from the catalytic pocket and acts as the docking site for signaling proteins [14]. The current data support this model. We showed that the deletion of the entire C-terminus abolished RON or RON160-mediated cell growth, shape change, and migration. Moreover, it eliminated the oncogenic potentials of RON160 acquired from the deletion of the first IPT domain. Thus, the entire C-terminus is a vital component of RON not only for structural integrity, but also for biological activities. Understanding the roles of the C-terminus should help us to gain insight into the mechanisms by which RON or RON160 exerts its activities relevant to cancer progression.

## Competing interests

The authors declare that they have no competing interests.

## Authors' contributions

YL and HPY performed experiments. MHW designed the study, performed certain experiments, and wrote the manuscript. All authors reviewed and approved the final manuscript for submission and publication.
